# Computer-Aided Cobb Measurement Based on Automatic Detection of Vertebral Slopes Using Deep Neural Network

**DOI:** 10.1155/2017/9083916

**Published:** 2017-10-03

**Authors:** Junhua Zhang, Hongjian Li, Liang Lv, Yufeng Zhang

**Affiliations:** ^1^Department of Electronic Engineering, Yunnan University, Kunming 650091, China; ^2^Department of Orthopedics, The First People's Hospital of Yunnan Province, Kunming 650032, China

## Abstract

**Objective:**

To develop a computer-aided method that reduces the variability of Cobb angle measurement for scoliosis assessment.

**Methods:**

A deep neural network (DNN) was trained with vertebral patches extracted from spinal model radiographs. The Cobb angle of the spinal curve was calculated automatically from the vertebral slopes predicted by the DNN. Sixty-five in vivo radiographs and 40 model radiographs were analyzed. An experienced surgeon performed manual measurements on the aforementioned radiographs. Two examiners used both the proposed and the manual measurement methods to analyze the aforementioned radiographs.

**Results:**

For model radiographs, the intraclass correlation coefficients were greater than 0.98, and the mean absolute differences were less than 3°. This indicates that the proposed system showed high repeatability for measurements of model radiographs. For the in vivo radiographs, the reliabilities were lower than those from the model radiographs, and the differences between the computer-aided measurement and the manual measurement by the surgeon were higher than 5°.

**Conclusion:**

The variability of Cobb angle measurements can be reduced if the DNN system is trained with enough vertebral patches. Training data of in vivo radiographs must be included to improve the performance of DNN.

**Significance:**

Vertebral slopes can be predicted by DNN. The computer-aided system can be used to perform automatic measurements of Cobb angle, which is used to make reliable and objective assessments of scoliosis.

## 1. Introduction

**Figure 1:**
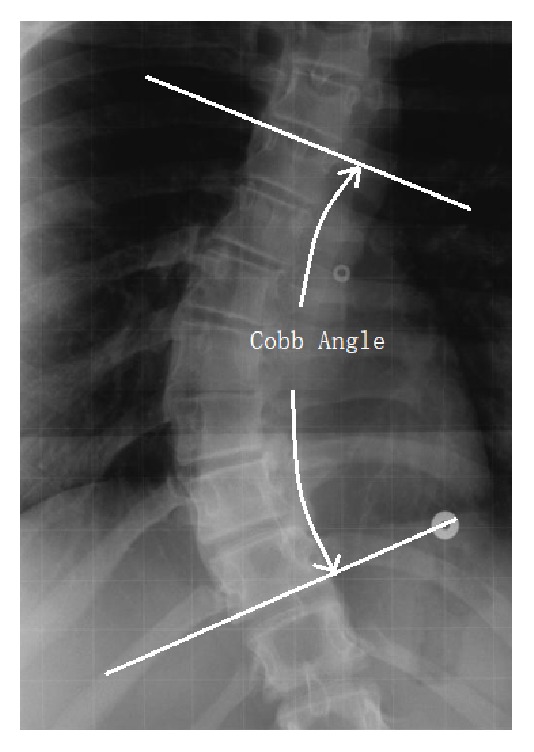
Cobb angle measurement.

Scoliosis is a spinal deformity in which the spine shows a lateral curvature in the frontal plane [1]. For scoliosis assessment, X-ray is the most economical imaging modality till date. The Cobb angle method [2] is the gold standard to assess curve severity in posteroanterior (PA) radiographs. To measure the Cobb angle, the most tilted vertebrae (end-vertebrae) at the top and bottom of the spinal curve are identified. As shown in Figure 1, the Cobb angle can be measured manually by determining the angle between the lines, respectively, drawn along the upper endplate of the superior end-vertebra and the lower endplate of the inferior end-vertebra. A patient's physiologic maturity, curve severity, and chances of progression are taken into consideration before making any treatment decisions, such as observation, bracing, and surgery. In general, an increase of 5° or more between two successive radiographs is considered indicative of curve progression. Therefore, the measuring procedure must be accurate and reproducible for correct diagnosis. Manual Cobb angle measurement requires experience and judgment. There could be errors in Cobb angle measurement if the surgeon selects different end-vertebrae and estimates different vertebrae slopes. Even when the same end-vertebrae are selected, Cobb angle measurements may vary by up to 5° for the same observer and by 7° for different observers [3, 4].

In recent studies, scientists have developed computer-aided methods to improve the repeatability of Cobb angle measurements. Zhang et al. [5] developed a computerized method based on the technique of Hough transform. This method detects vertebral endplates, which are then used to automatically calculate the Cobb angle. In this method, false detection might occur if a vertebra tilts by more than 45°. To facilitate the identification of end-vertebrae and to eliminate observer variations, Anitha and Prabhu [6] proposed listing the horizontal inclinations of all vertebrae in terms of slopes by using active contour models and morphological operators. Okashi et al. [7] developed an automatic method for spine segmentation and curvature quantification from poor-quality X-ray images of mice. They also investigated the applicability of their method to the X-ray image of a human spine, and they believed that their method could be a good solution for low-quality human X-ray images. Anitha et al. [8] proposed an automated system that extracted vertebral endplates using a customized filter that combined anisotropic, sigmoid, and differential filters. Samuvel et al. [9] proposed a mask-based segmentation algorithm for automatic Cobb angle measurement. The aforementioned algorithm and the manual method were used for measuring the Cobb angle in six cases. There was a difference of about 4° between the results obtained by the algorithmic method and those obtained by the manual method. Kusuma et al. [10] estimated vertebral locations by performing the template matching technique on the subdivisions of radiographs; the technique was based on the sum of squared differences. Then, they estimated the spinal curvature by polynomial curve fitting. In their method, the final measurement was affected by the polynomial order and the number of subdivisions. They achieved an average accuracy of 86.01%. Wibowo and Sardjono [11] used a modified top-hat filtering method for preprocessing and the gradient vector flow snake for the automatic determination of spinal curvature. Toan et al. [12] performed Cobb angle measurement by a semiautomatic method based on a deformable model of fuzzy spatial relations. Langensiepen et al. [13] presented a systematic review of the various techniques used to carry out Cobb angle measurement.

In this study, we proposed a computer-aided Cobb angle measurement method that automatically determined the slopes of vertebrae by using a deep neural network (DNN). DNNs with good generalization capability show promise in the field of machine learning. After being trained using vertebral patches, the DNN could be used to predict vertebral slopes in real time.

## 2. Materials and Methods

### 2.1. Radiographic Data

In this study, we used a spine model to generate the training set. This model included 12 thoracic vertebrae and five lumbar vertebrae. This model was made in a high-resolution imaging medium (Sawbones, Vashon, WA, USA) that could be used to produce a realistic image in radiographic environments as real bones. A total of 275 PA radiographs of the spine model in different poses were collected with vertebral slopes in the range of 5°–50° (mean: 15°). Lead markers that could be detected in radiographs were attached to the pedicles of each vertebra in the model. The detected markers were used to calculate the slope of a vertebra; this value was considered as the ground truth slope of that vertebra. In addition, we used the radiographs of 65 patients (51 girls, 14 boys; age: 12.5 ± 3.6 years) with idiopathic scoliosis. The selection criteria were as follows: (1) diagnosis of idiopathic scoliosis; (2) age between 9 and 18 years; (3) no prior spine surgery; and (4) visibility of pelvis, 12 thoracic vertebrae (numbered T1–T12), and 5 lumbar vertebrae (numbered L1–L5) in the radiographs. In this study, we excluded patients who had other musculoskeletal or neurological disorders. Informed consent was obtained from all patients/parents. This study received ethical approval from the local ethics board.

An image preprocessing protocol was applied to all the radiographs. In this protocol, the spinal radiograph was cropped from T1 to L5 and the images were resized to a standard height of 1000 pixels. Out of the 275 radiographs of the spine model, we randomly selected 235 radiographs to generate training patches for DNN training. Each selected radiograph showed 12 thoracic vertebrae and five lumbar vertebrae. For each vertebra in the radiograph, we randomly extracted 100 patches of 150 × 150 pixels. These patches were downsampled thrice to obtain patches of 50 × 50 pixels; these were represented by vectors of 2500 gray-level values. We conducted DNN training using 399500 patches (235 radiographs, each containing 17 vertebrae; 100 patches were extracted for each vertebra). The performance of the DNN system was tested on 105 radiographs, which included the 65 radiographs of patients and the remaining 40 radiographs of the spine model.

### 2.2. DNN for Estimation of Vertebral Slope

**Figure 2:**
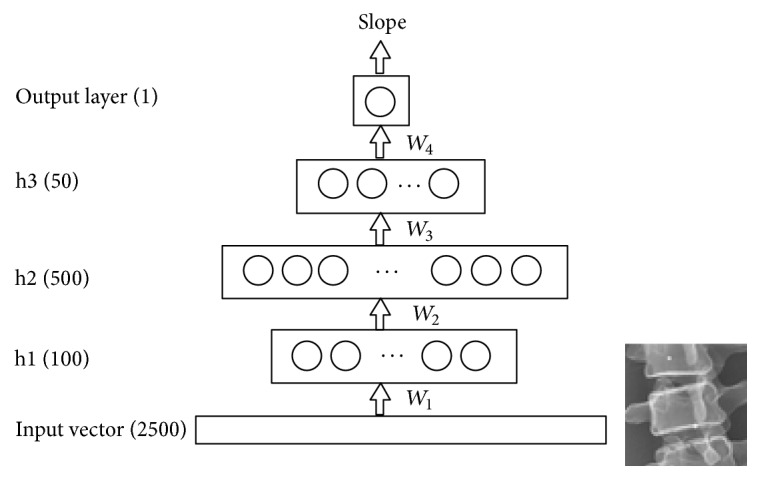
Architecture of the proposed network.

A DNN was trained to predict the slope of a vertebra as a function of a patch (*P*). As shown in Figure 2, the network architecture comprised three hidden layers of neurons: h1, h2, and h3. The first hidden layer h1 had 100 neurons whose inputs were a patch of 50 × 50 pixels (i.e., 2500 input units). The second hidden layer h2 had 500 neurons, while the third hidden layer h3 had 50 neurons. In the output layer and the first hidden layer (h1), the neurons had linear activation functions. In the other hidden layers, neurons had hyperbolic tangent functions. The corresponding function of the DNN was a vector mapping *A*(*P*): **R**^2500^→**R**^1^, where

(1) AP=b4+W4tanh⁡b3+W3tanh⁡b2+W2W1P−b1.

Here *W*_1_,…, *W*_4_ were the weight matrices of each layer, and *b*_1_,…, *b*_4_ were the bias vectors. The first hidden layer acted as a low-pass filter for multidimensionality reduction based on principal components analysis (PCA). Therefore, *W*_1_ comprised the first 100 eigenvectors of the patches covariance matrix, representing the PCA basis, and* b*_1_ was the mean of data patches. The second hidden layer h2 received the latent variable of the PCA as an input. To properly initialize the weights of the network, we pretrained the DNN using autoencoders with a learning rate of 0.1 for each layer. The DNN was then fine-tuned using error backpropagation algorithm. To train the DNN, we used the Python deep learning library Keras with the Theano backend. After the training procedure, the DNN rapidly predicted the slope of any vertebral patch because the DNN performed only simple matrix operations for computing the slope.

### 2.3. Cobb Angle Measurement Based on Estimated Vertebral Slopes

**Figure 3:**
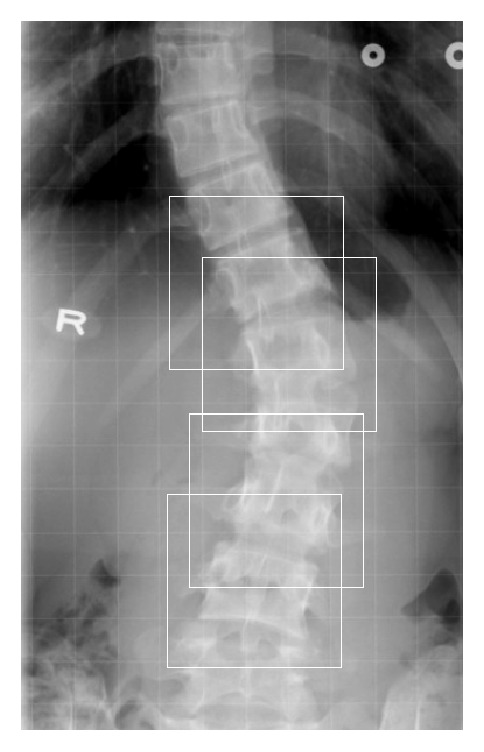
Vertebral patches assigned by a user.

In the proposed method, the DNN was used to estimate the slopes of vertebrae, and the Cobb angle was calculated from these slope values. The proposed method was not full automatic as the user had to assign vertebral patches. In the PA radiograph, for a spinal curve to be measured, the user selected several vertebrae that contained the upper and lower end-vertebrae by clicking them with the mouse. Then, a rectangle of 150 × 150 pixels was created for each selected vertebra, as shown in Figure 3, and sent to the DNN to obtain the slope of that vertebra. The two vertebrae with the maximum absolute slopes tilting in opposite directions to the horizontal were detected automatically. The Cobb angle was then calculated automatically as the sum of the absolute values of the two slope angles.

### 2.4. Performance Analysis

The proposed method was tested and compared with the manual measurement method to evaluate its performance. An orthopedic surgeon (S) specialized in scoliosis with 25 years of experience manually measured the Cobb angle in 105 testing radiographs. The results obtained from the surgeon were considered as true values. Then, two examiners measured the Cobb angle in each radiograph using the software developed in this study. Both of them measured the Cobb angle twice over a period of three weeks. Examiner 1 (E1) had worked in a scoliosis clinic for 21 years. Examiner 2 (E2) was the software developer with no clinical experience of measuring the Cobb angle manually. The two examiners were asked to manually measure the Cobb angle in each radiograph twice over a period of one week. The examiners' manual measurements were performed 6 months after their computer-aided measurements. All measurements on the same radiograph were performed on the same curve, although some spines had multiple curves. Statistical analyses were performed using SPSS 16.0 (SPSS Inc., Chicago, IL, USA) software. The intraclass correlation coefficient (ICC, value between 0 and 1) with 95% confidence interval (CI) was used to evaluate the reliability [14]. For error analysis, the mean absolute difference (MAD) of two measurements was also calculated.

## 3. Results and Discussion

The intraobserver reliability of the proposed method was assessed for each examiner and was compared with that of the manual measurement.  Table 1 presents the results of intraobserver analyses. For the measurements obtained by the proposed method, all the intraobserver analyses produced ICC > 0.9 with 95% CI between 0.811 and 0.991 and MAD < 5°. The results of model radiographs showed that the proposed method had high intraobserver reliability (ICC > 0.98, MAD < 3°). Compared to the model radiographs, the proposed method showed lower intraobserver reliability for the results of in vivo radiographs. This was mainly because all the training patches were sampled from model radiographs.

Table 2 presents the results of interobserver reliability analyses. By using the proposed method, the interobserver reliability for the model radiographs was high; ICC ≥ 0.98 with 95% CI between 0.962 and 0.990, and MAD ≤ 3°. These results indicate that the automatic method was consistent regardless of the examiners' experience. However, for the in vivo radiographs, the interobserver analyses produced ICC < 0.9 with 95% CI between 0.689 and 0.973. In the first trial, the MAD was more than 5°. Therefore, in vivo radiographs should be added in the training process to improve the system performance.

For the model radiographs, the intraobserver and interobserver reliabilities of the proposed method were higher than those of the manual method. For the in vivo radiographs, by using the proposed method the intraobserver reliability of E1 was close to that of E2. For the in vivo radiographs examined by E2, the intraobserver reliability of the proposed method was close to that of manual measurement, while for the in vivo radiographs examined by E1 the intraobserver reliability of the proposed method was lower than that of manual measurement. These results indicate that the reliability of manual measurements depends on the relevant experience of the examiner. The proposed method can outperform manual measurements if the DNN is trained adequately.

In previous studies, the reliabilities of computer-aided methods [5, 6, 8] were less than 3° for the Cobb angle measurements. For the model radiographs, the results of the proposed method are comparable to those reported in previous studies (i.e., ≤3°). However, for the in vivo radiographs, the reliability of the DNN system was less than the reliability of methods described in previous studies. Therefore, the training patches should be sampled out from both model and in vivo radiographs to train the DNN adequately.

To evaluate the validity of the proposed method, automatic measurements performed by two examiners were compared with the manual measurement performed by the surgeon.  Table 3 shows the results. For the model radiographs, both examiners produced ICC > 0.9 with 95% CI between 0.815 and 0.962. The MAD between the automatic and manual measurements was less than 5°. These results indicate that the automatic method showed good agreement with the manual measurement method. Even an examiner with little experience could obtain similar results with the automatic method. However, for the in vivo radiographs, all the MAD values were greater than 5°. Therefore, the DNN system should be trained with adequate number of patches obtained from in vivo radiographs. In this study, the DNN system was only trained with patches obtained from model radiographs.

To explore the effect of network architecture, we trained three networks containing three different numbers of neurons in the second and third hidden layers (h2 and h3): h2 had 500 neurons and h3 had 50 neurons; h2 had 800 neurons and h3 had 100 neurons; and h2 had 500 neurons and h3 had 250 neurons. We collected 680 vertebral patches from 40 model radiographs (i.e., 40 × 17 = 680) with known ground truth slopes to test the three networks. The average errors of the vertebral slope tested with the three networks were 2.0°, 1.9°, and 2.0°, respectively. Therefore, in the proposed system we chose the simplest network with h2 and h3 having 500 and 50 neurons, respectively.

In clinical practice, one of the major sources of Cobb measurement errors is manually drawing lines across the endplates of the end-vertebrae. In this study, we developed a computer-aided system to reduce the human judgment errors in Cobb angle measurements. In the developed system, the user was required to only select the patches. This was the only source of variability in the developed system. The computer-aided system automatically calculated the Cobb angle from the patches assigned by the user. For the model radiographs, the intraobserver and interobserver analyses showed excellent reliability of the proposed method (ICC ≥ 0.98) with MAD ≤ 3°; this was lower than the 5° threshold of changes that could influence treatment decisions. Because the DNN was trained with only model radiographs, the system performance was not reliable for in vivo radiographs. The system performance could be improved further by training the DNN with sufficient number of in vivo radiographs.

## 4. Conclusion

Cobb angle measurement errors usually occur when lines are drawn manually across the endplates of end-vertebrae. In the proposed method, the slopes of vertebrae were automatically predicted using a DNN that was trained using model radiographs. The intraobserver and interobserver analyses indicated that the measurement variation was lower than the threshold of changes that influence treatment decisions. Although the user is required to assign vertebral patches in the proposed method, the user needs fewer skills to perform this task. By using the trained DNN, the measurement could be completed in real time after the user assignment of vertebral patches. Although we need to collect more in vivo data to improve the performance of the proposed system, the system has the potential to reduce the variation in scoliosis assessment. Further studies must be conducted to determine whether the proposed method can be safely used in clinical assessment of scoliosis.

## Figures and Tables

**Table 1:** Intraobserver analyses.

	ICC (95% CI)		Mean absolute difference	
	From model	From patients	From model	From patients
Proposed measurement				
E1	0.986 (0.976, 0.991)	0.901 (0.812, 0.962)	2.6°	4.6°
E2	0.985 (0.974, 0.990)	0.908 (0.811, 0.970)	2.6°	4.4°
Manual measurement				
E1	0.964 (0.902, 0.985)	0.953 (0.893, 0.981)	3.6°	3.7°
E2	0.937 (0.851, 0.979)	0.908 (0.809, 0.970)	4.1°	4.5°

**Table 2:** Interobserver analyses.

	ICC (95% CI)		Mean absolute difference	
	From model	From patients	From model	From patients
Proposed measurement				
1st	0.980 (0.962, 0.988)	0.874 (0.689, 0.960)	3.0°	5.1°
2nd	0.981 (0.964, 0.990)	0.889 (0.736, 0.973)	2.9°	4.9°
Manual measurement				
1st	0.870 (0.694, 0.955)	0.862 (0.659, 0.945)	5.1°	5.3°
2nd	0.892 (0.751, 0.983)	0.878 (0.697, 0.967)	4.8°	5.0°

**Table 3:** Comparison between automatic and manual measurements.

	ICC (95% CI)		Mean absolute difference	
	From model	From patients	From model	From patients
E1 versus S	0.912 (0.815, 0.962)	0.835 (0.701, 0.914)	4.4°	5.4°
E2 versus S	0.915 (0.901, 0.927)	0.771 (0.602, 0.874)	4.9°	6.6°
